# 876. The Influence of Race and Social Determinants of Health on Mortality in Burn Injury Patients

**DOI:** 10.1093/jbcr/irag033.362

**Published:** 2026-04-06

**Authors:** Joshua E Lewis, Olivia Anga, Jarrell Patterson, Blancheneige M Beohon, Chrishaun Alexander, Raven Hollis, Erin Jackson, Calah Burros, Brianna L Gilner, Bryan S Torres, Oyetokunbo Ibidapo-Obe

**Affiliations:** Indiana University Health, Galveston, TX; University of St. Thomas, Houston, TX; Brody School of Medicine East Carolina University, Greenville, NC; University of Texas Medical Branch, Galveston, TX; Morehouse School of Medicine, Atlanta, GA; University of Houston College of Medicine, Houston, TX; Prairie View A&M University, Prairie View, TX; Prairie View A&M University, Prairie View, TX; Whiddon College of Medicine at the University of South Alabama, Mobile, AL; Tulane School of Medicine, New Orleans, LA; Memorial Hermann, Houston, TX

## Abstract

**Introduction:**

Burn injuries continue to contribute significantly to morbidity and mortality globally, with previous studies highlight disparities influenced by both clinical and social factors. However, limited research has been conducted on the influence on social determinants of health and race on mortality rates. This study investigates the impact of race and SDOH, including unemployment, low health literacy, homelessness, and family dynamics, on mortality following burn injuries.

**Methods:**

This retrospective cohort study analyzed data from 147 802 551 patients across 109 healthcare organizations as of July 18, 2025. Patients with burn injuries identified by ICD-10 codes T20–T32 were included. Two stratifications were performed: first, patients with burn injury and specific SDOH were compared to a control group without these SDOH; second, patients were stratified by race and compared to White patients. Propensity score matching controlled for demographic variables and comorbidities. Mortality risk ratios (RRs), 95% confidence intervals (CIs), and *p*-values were calculated at 3-, 6-, and 12-months post-injury. Statistical significance was defined as *p*<.05.

**Results:**

Social determinants of health (SDOH) significantly increased mortality risk after burn injury. At 3 months post-burn, unemployment was associated with the highest risk of mortality (RR 3.052, *p*=.0013), followed by education and literacy challenges (RR 2.187, *p*=.0261), housing and economic instability (RR 2.018, *p*<.0001), and family dynamics issues (RR 1.912, *p*=.0002). These elevated risks persisted consistently at 6 and 12 months. Additionally, there was evident of racial differences. Black patients had a significantly higher risk of mortality at 3 months (RR 1.309, *p*<.0001), Hispanic patients also demonstrated significantly increased mortality risk at 3 months (RR 1.812, *p*=.0004), American Indian or Native Hawaiian (“Other races”) had significantly increased mortality risk at 3 months. In contrast, Asian patients did not show a significant increase at 3 months (RR 0.974, *p*=.7294) but had significantly lower mortality risk at 6 months (RR 0.802, *p*=.0003) and 12 months (RR 0.780, *p*<.0001). Similar trends for Hispanic, Black, and other races were persistent across six and 12 months.

**Conclusions:**

This comprehensive study demonstrates that both social determinants of health and race can influence mortality risk following burn injuries. Targeted interventions addressing these disparities are critical to improving outcomes and achieving health equity in burn care.

**Applicability of Research to Practice:**

Recognizing and integrating SDOH and racial disparities into burn care protocols can improve risk and resource allocation. Healthcare providers and systems should prioritize social risk screening and culturally competent interventions to reduce preventable mortality in vulnerable burn populations.

**Funding for the study:**

N/A.

